# Galanin analogs prevent mortality from seizure-induced respiratory arrest in mice

**DOI:** 10.3389/fncir.2022.901334

**Published:** 2022-08-16

**Authors:** Ryley Collard, Miriam C. Aziz, Kevin Rapp, Connor Cutshall, Evalien Duyvesteyn, Cameron S. Metcalf

**Affiliations:** ^1^Department of Pharmacology and Toxicology, University of Utah, Salt Lake City, UT, United States; ^2^Epilepsy Therapy Screening Program Contract Site, Department of Pharmacology and Toxicology, University of Utah, Salt Lake City, UT, United States

**Keywords:** animal models, respiratory arrest, SUDEP, neuropeptide, epilepsy

## Abstract

**Objective:**

Sudden Unexpected Death in Epilepsy (SUDEP) accounts for 20% of mortality in those with recurrent seizures. While risk factors, monitoring systems, and standard practices are in place, the pathophysiology of SUDEP is still not well understood. Better knowledge of SUDEP and its potential mechanisms of action is crucial to reducing risk in this patient population and developing potential treatment options. Clinical studies and animal models of SUDEP suggest that diminished post-ictal respiratory control may be the dominant mechanism contributing to mortality. Recently, it was demonstrated that the depletion of the neuropeptide galanin in the amygdala occurs in human SUDEP. The amygdala plays a key role in the central integration of respiratory signaling; the depletion of galanin may represent a critical change that predisposes individuals to SUDEP.

**Materials and methods:**

To evaluate the impact of enhancing galaninergic signaling to potentially protect against SUDEP, we studied seizure-induced respiratory arrest (S-IRA) following central (intracerebroventricular, intra-amygdala) and systemic (intraperitoneal, subcutaneous) administration of galanin analogs. Seizure naïve and seizure experienced (fully kindled) mice were tested.

**Results:**

Central and systemically administered galanin analogs protect against S-IRA in naïve C57Bl/6J mice. Differential efficacy between receptor subtype-selective analogs varied based on the route of administration. Sub-chronic systemic administration at doses that reduced 6 Hz seizures also protected against S-IRA. Acute treatment benefits also extended to fully kindled mice experiencing tonic extension.

**Significance:**

These data demonstrate that galanin analogs may be protective against post-ictal respiratory collapse.

## Highlights

– Central and systemic galanin analogs prevent seizure-induced respiratory arrest.
– Efficacy was observed in three separate mouse strains under various experimental conditions.
– Sub-chronic administration demonstrated galanin analog protection against respiratory arrest.
– Acute systemic administration also conferred protection against respiratory arrest following tonic extension in fully kindled mice.
– Galanin analogs may represent a novel potential therapy in SUDEP-susceptible individuals.

## Introduction

Epilepsy can lead to a variety of serious complications and is associated with a reduction in life expectancy. One of the leading causes of mortality in those with recurrent seizures is an event known as Sudden Unexpected Death in Epilepsy (SUDEP) (Devinsky et al., 2016; Dlouhy et al., 2016; DeGiorgio et al., 2017; Jones and Thomas, 2017; Michalak et al., 2017). A pivotal clinical study demonstrated that post-seizure (post-ictal) apnea precedes asystole (Ryvlin et al., 2013), suggesting that respiratory control may be a major mechanism in SUDEP. Further, clinical observations and mouse models suggest that respiratory neurocircuitry is altered in individuals susceptible to early mortality (Seyal et al., 2010; Mueller et al., 2014; Lacuey et al., 2018; Kuo et al., 2019; Vilella et al., 2019a). Death after seizures may result from a combination of poor respiratory responses and failure in normal arousal mechanisms that promote breathing (Tomson et al., 2008; Tolstykh and Cavazos, 2013; Devinsky et al., 2016; Dlouhy et al., 2016; Vilella et al., 2019a,b).

Generalized tonic-clonic seizures (GTCS) are a major risk factor for SUDEP and may lead to hypoxia, hypercapnia, failed arousal mechanisms, and thus an inability to recover breathing following seizures (Monte et al., 2007; Nashef et al., 2007; Devinsky et al., 2016; Dlouhy et al., 2016). A GTCS can spread to other brain regions, including those involved in breathing. Several forebrain and brainstem nuclei may regulate breathing following seizures. One important area of interest is implicated in post-ictal respiration is the amygdala. Apnea results when seizures spread to the amygdala, and amygdala lesions reduce seizure-induced respiratory arrest (S-IRA) in mice (Marincovich et al., 2019; Nobis et al., 2019; Rhone et al., 2020). Moreover, the amygdala has direct connections with brainstem breathing control sites (Saha et al., 2000; Dong and Swanson, 2006). The amygdala is enriched in neuropeptides that modulate neuronal activity and can be depleted following seizures (Somani et al., 2020). Interestingly, depletion of the neuropeptide galanin in the amygdala occurs in human SUDEP (Somani et al., 2020). Galanin is an anticonvulsant neuropeptide activated under conditions of enhanced neural activity (Lang et al., 2015) and plays a critical role in responses to respiratory stressors (Stornetta et al., 2009; Shi et al., 2017). Further, galanin is robustly upregulated after various insults such as nerve injury and seizures (Mazarati et al., 2000; Liu and Hokfelt, 2002; Lundstrom et al., 2005). Similarly, galanin is increased in the amygdala following seizures (Christiansen and Woldbye, 2010). Therefore, the amygdala is a critical relay point between seizures and downstream breathing control following seizures, and the depletion of galanin may represent a critical pathophysiologic change predisposing some individuals to SUDEP. It may also be that enhancement of galanin signaling in the amygdala protects against respiratory collapse following seizures.

There remains no evidence-based treatment to prevent SUDEP. The mainstay of management has been addressing modifiable risk factors such as ensuring medication adherence, but with few results (Tomson et al., 2008; Lhatoo et al., 2015; Devinsky et al., 2016; Dlouhy et al., 2016). Novel therapies may therefore prove beneficial if they offer benefits targeted explicitly to SUDEP risk, in addition to any potential benefits of reducing seizure burden. Therefore, the purpose of this study was to use rodent models to demonstrate how galanin analogs may prevent mortality in mice experiencing S-IRA. Respiratory arrest, arising following tonic extension (TE) elicited in mice, has been used as a model of SUDEP and recapitulates apnea following GTCS (Purnell et al., 2017; Kruse et al., 2019). Further, this model offers the opportunity to screen compounds with acute and chronic effects at preventing S-IRA. These studies described herein will therefore demonstrate the potential utility of galanin analogs in restoring respiration after seizures in naïve and seizure-experienced mice.

## Materials and methods

### Animals

Three different mouse strains were used: male C57BI/6J (5–6 weeks old, Jackson Laboratory, Bar Harbor, ME, United States), male CD-1 (5–7 weeks old, Charles River Laboratories, Kingston, NY, United States), and male CF-1 (5–7 weeks old, Charles River Lab, Kingston, NY, United States). Animals were allowed free access to food and water, except during testing periods. Prior to testing, animals were allowed 1 week to acclimate to housing conditions. All mice were housed in plastic cages in rooms with controlled humidity, ventilation, and lighting (12 h on–12 h off). The animals were housed and fed in a manner consistent with the recommendations in the “Guide for Care and Use of Laboratory Animals” (National Research Council). Housing, handling, and testing was performed in accordance with Public Health Service policy guidelines and a protocol approved by the Institutional Animal Care and Use Committee of the University of Utah.

### Compound preparation/administration

A total of 810–2 (Gal_2_-preffering) and 505–5 (Gal_1_-preferring) (molecular weights 2,124 and 2,113 g/mol, respectively) (White et al., 2009; Bialer et al., 2013, 2015, 2017; White, 2017) was synthesized by PolyPeptide Laboratories (San Diego, CA United States). A total of 810–2 was dissolved in a vehicle (VEH) solution of 2% (central) or 20% (systemic) hydroxy propyl beta cyclodextrin (HPβCD; Sigma, St. Louis, MO, United States) and 3.75% D-mannitol in acetate buffer [0.1 M acetic acid (from glacial acetic acid stock; Sigma, St. Louis, MO, United States); 0.1 M sodium acetate (Sigma, St. Louis, MO, United States), pH 4.5]. A total of 505–5 was dissolved in a VEH solution of 1% Tween 20 (Sigma, St. Louis, MO, United States) in 0.9% NaCl. Compounds were administered by intraperitoneal (IP), intracerebroventricular (ICV; free-hand injection), or intra-amygdala (IA; 1–2 weeks post unilateral amygdala cannulation). For ICV and IA administration of 810–2, the vehicle solution was 2% HPβCD in water. Separate experiments were also conducted for administration of galanin analogs by subcutaneous (SC) sub-chronic (14-day) administration. A total of 810–2 and 505–5 were prepared in 50:50 dimethylsulfoxide (DMSO): water at concentrations of 67.2 and 16.8 mg/ml (respectively), and placed in implantable mini-pumps (Alzet, model 1002). For SC administration in pumps, larger concentrations (e.g., 16.8, 67.2 mg/ml) are beyond solubility limitations using the HPβCD-acetate buffer vehicle (used for IP, ICV, and IA). Therefore, the peptides were solubilized in DMSO: H_2_O and only a small total volume (∼100 μl) was used for each pump). By contrast, volumes injected for intraperitoneal administration are much greater and the larger relative amounts of DMSO would produce untoward effects.

### Surgical implantation of unilateral amygdala cannulas

Procedures were similar to those described previously for implantation of unilateral cannulas for drug administration (West et al., 2022). Buprenorphine (0.01–0.2 mg/kg) was administered 1 h prior to surgery. Isoflurane (2–5% in O_2_) was used for anesthesia, and mice were placed in a stereotaxic apparatus. A Dremel drill was used to drill a single hole over the right hemisphere followed by placement of a 22-gauge cannula above the dura mater (AP −1.2, ML 3.3). This guide cannula was glued in place using dental acrylic. Antibiotic ointment was applied to the surface around the head cap and penicillin (60000 units, SC) was administered. Mice were then allowed to recover in their home cages (singly housed) for ∼1 week.

### Surgical implantation of osmotic minipumps

Alzet minipumps were prepared and immersed in saline overnight. Under isoflurane anesthesia (2–5% in O_2_), as described above, a small incision was made between the scapulae (shaved prior to incision) followed by clearance of subcutaneous fascia with forceps. Minipumps were placed in the SC space and the incision sutured with surgical silk. Antibiotic ointment was placed around the incision and mice were allowed to recover. Mice were monitored daily for the duration of the 14-day treatment.

### Electrical seizure induction and post-stimulation monitoring

#### Maximal electroshock seizure model

Maximal electroshock seizure has been used as a model of GTCS for preclinical pharmacology studies routinely for many years (Loscher, 2017). Furthermore, TE following MES stimulation is followed by apnea and death in some mouse strains and has been used as a model of S-IRA (Purnell et al., 2017). Prior to testing, tetracaine (0.5% in saline) was applied to the corneas. MES seizures were induced using a 50 mA current (0.2 s duration) *via* corneal electrode (Barton et al., 2001, 2003). This stimulation intensity produces TE in nearly all mice tested. Following stimulation, mice were observed for the presence of TE and the resumption of post-ictal breathing. Mice not displaying TE were considered protected.

#### 6 Hz seizure induction

This seizure assay has been used as a model of preclinical pharmacology studies in mice (Barton et al., 2001) and was used in these studies to confirm efficacy observed following treatment with galanin analogs (Bulaj et al., 2008; White et al., 2009; Metcalf et al., 2017a). Seizures were induced using the 32 mA stimulus intensity (3 s, 6 Hz, 32 mA) *via* corneal electrodes. Prior to testing, tetracaine (0.5% in saline) was applied to the corneas. Seizure induction is associated with characteristic behaviors including jaw and forelimb clonus with or without loss of righting and rear limb clonus. Animals not displaying any of these behaviors were considered protected.

#### Corneal kindling

The mouse corneal kindling model was used in CF-1 and C57Bl/6J mice. Mice receive twice daily corneal stimulation (60 Hz, 3 s, 5 days/week) (3 mA–CF-1; 1.5 mA–C57Bl/6). Seizures are scored using the Racine scale (Racine, 1972) and progress during kindling from mild motor behaviors (e.g., jaw clonus) to generalized seizures (bilateral forelimb clonus, rearing and falling). When five consecutive stage five (generalized seizure with loss of righting reflex) seizures are observed, animals are considered fully kindled. Drug administration studies were performed on fully kindled mice, where MES was applied after drug treatment. TE following MES was evaluated similarly to that described above for naïve animals.

### Respiratory monitoring

CF-1 mice subjected to MES were also monitored for cardiorespiratory activity before and following seizures using a MouseOx (Starr Life Sciences) monitoring system. While other strains of mice (e.g., CD-1, C57Bl/6) experience a high mortality following TE, CF-1 mice survive this stimulation. This mouse strain was included for respiratory evaluation so that recordings could be obtained prior to and following seizures. On the day prior to testing, mice were anesthetized with 2–5% isoflurane and the neck region shaved with a surgical razor. On the following day, prior to seizure induction, mice were acclimated to pulse oximetry collars for ∼15 min followed by a baseline recording session (5 min). A plastic sham collar (not connected to monitoring hardware) was placed initially, followed by oximetry collars and baseline recording sessions. After the baseline recording, mice were subjected to MES stimulation followed by a post-stimulation recording session (5 min). Oximetry data were extracted as text files and transferred to Microsoft Excel databases for data analysis. A 30-s recording epoch was selected from each recording session and heart rate, respiratory rate, and oxygen saturation (SpO_2_) values averaged over this period.

### Intraperitoneal administration

A total of 810–2 and 505–5 were administered to mice 1 h prior to MES stimulation This time point was selected based on previous studies demonstrating peak efficacy for these analogs in the mouse 6 Hz assay 1 h following IP administration (Bulaj et al., 2008; White et al., 2009). A total of 810–2 was administered at doses of 8, and 16 mg/kg IP (*N* = 10-24 per group), doses were selected based on previous *in vivo* efficacy studies for this compound (Bialer et al., 2015; Metcalf et al., 2017b). A total of 505–5 was administered at doses 2, and 4 mg/kg IP (*N* = 9–24 per group). Doses for each compound were selected based on previous *in vivo* efficacy studies (White et al., 2009; Bialer et al., 2013, 2015; Metcalf et al., 2017b). A separate group of mice were treated with VEH (*N* = 37), IP administration, 1 h pre-treatment time.

### Central administration

Mice received a single administration centrally of 810–2, 505–5, or VEH. A 5 μl injection volume was used for all central injection studies, and mice only received one injection before being tested and euthanized. Doses of 1–4 nmol were used for intracerebroventricular (ICV) administration whereas a larger dose range was used for IA treatment. For ICV administration a 10 μl Hamilton syringe with a 25-gauge needle was used for administration. Mice were placed under gentle restraint and a free-hand injection was made through the skull surface and into the lateral ventricular space. By contrast, for IA injection studies, mice were implanted 1–2 weeks prior with cannulae centered unilaterally over the amygdala and an injection needle was used to administer compounds over a 30 s period. Initial low doses of 810–2 were selected (0.02–2 nmol) to evaluate tolerability to injection of peptides in this brain region. Following a maximal dose (4.7 nmol) was used, comparable to that used for ICV studies. Treatments occurred 15 min prior to testing for all central injections. For the purposes of these studies, validation of ICV or IA injection was not included. We have previously used ICV free-hand injections for central administration of neuropeptides (Lee et al., 2009; Green et al., 2011; Platt et al., 2012). Similarly, the methods described for IA administration utilize a guide cannula and the same surgical procedures, coordinates, and injection techniques used in our lab as those described for central administration of kainic acid to the amygdala (West et al., 2022).

### Statistical analysis

The data was presented as means ± standard error. A comparison between two means was performed using a Student’s *t*-test, and multiple comparisons were made using a one-way ANOVA followed by a Newman-Keuls or a Dunnett’s test for *a posteriori* analysis of the difference between group means. A Fisher’s exact test was used to compare numbers of mice protected and/or surviving between groups. Survival analyses were analyzed using a log-rank (Mantel-Cox) test. Results where *P* < 0.05 was considered significant.

## Results

### Galanin analogs reduce seizure-induced respiratory arrest in mice: Differential effects of route of administration and analog

Galanin analogs 810–2 and 505–5 were administered to C57Bl/6J mice using different routes of administration, followed by MES stimulation for evaluation of S-IRA. First, each analog was administered 1 h prior to testing using IP administration: 810–2 8,16 mg/kg, 505–5 (2, 4 mg/kg) vs. VEH. All mice tested demonstrated a characteristic TE following MES (Tedeschi et al., 1956; Loscher, 2011, 2017). Typically, breathing is absent or diminished during TE in all mice, and many mice die as a result, particularly for CD-1 and C57Bl/6J strains. Following corneal MES stimulation, all mice experienced tonic extension. As shown in Table 1, VEH treatment was associated with high mortality (62%, 23/37 mice died) following TE. Although systemic administration of 810–2 did not prevent TE, the analog decreased mortality following TE at a dose of 16 mg/kg (25%, 5/20 died; *P* < 0.05, Fisher’s exact test). By contrast, 505–5 did not prevent TE or reduce mortality at the doses tested. As higher doses of each analog are associated with untoward effects (sedation, lethargy), additional doses were not included. CD-1 mice respond similarly to testing, and both 505–5 and 810–2 significantly improve mortality following TE in these mice (505–5, 4 mg/kg IP, *P* < 0.05 vs. VEH, Fisher’s exact test; 810–2, 16 mg/kg, *P* < 0.05 vs. VEH, Fisher’s exact test; Supplementary Table 1).

**TABLE 1:** Systemic and central administration of galanin analogs: effect on (S-IRA) in C57B1/6J mice.

Compound	Mortality (# died/N) (%)		
	Systemic (IP)	Central (ICV)	Central (IA)
VEH	23/37 (62%)	13/16 (81%)	11/17 (65%)
505–5	2 mg/kg: 9/20 (45%) 4 mg/kg: 8/20 (40%)	1 nmol: 11/16 (69%) 2 nmol: 4/8 (50%) 4 nmol: 4/11* (36%)	4.7 nmol: 9/21 (43%)
810–2	8 mg/kg: 11/19 (58%) 16 mg/kg: 5/20* (25%)	1 nmol: 6/11 (55%) 2 nmol: (10/16) (63%) 4 nmol (11/16) (69%)	0.02 nmol: 6/7 (86%) 0.1 nmol: 8/8 (100%) 0.2 nmol: 9/9 (100%) 4.7 nmol: 6/23* (26%)

*P < 0.05, Fisher’s exact test vs. VEH. Routes of administration: IP (intraperitoneal), ICV (intracerebroventricular), IA (intra-amygdala). All mice tested experienced tonic extension.

Following systemic administration studies, ICV administration was performed. Previously, doses of 1–2 nmol (ICV) demonstrated efficacy against 6 Hz seizures for galanin analogs (Bulaj et al., 2008). Therefore, doses in this range were initially used for these studies. Similar to that observed following systemic administration, ICV VEH was associated with a high mortality rate (81%, 13/16) following TE. While the 810–2 did not prevent TE, and was without significant effect on mortality using the doses tested, 505–5 reduced mortality from S-IRA at the highest dose tested (4 nmol; 4/11, *P < 0.05 vs. VEH, Fisher’s exact test) without preventing TE. Next, using unilateral IA cannulas, galanin analogs were administered by direct injection in freely behaving mice. Initially, a dose of VEH was administered and showed a high mortality rate (65%; 11/17). Following several low doses of 810–2 were administered (0.02–0.2 nmol) to determine whether direct injection would produce untoward effects. As these concentrations were well-tolerated, a higher concentration (4.7 nmol) was administered for both 505–5 and 810–2. A total of 810–2 significantly reduced mortality (26%, 6/23; *P* < 0.05 vs. VEH, Fisher’s exact test) following TE, whereas 505–5 was not significantly effective (43%; 9/21). None of the doses tested prevented TE.

### The Gal_2_-preferring analog 810–2 prevents mortality from seizure-induced respiratory arrest following sub-chronic subcutaneous administration

We sought to determine whether sub-chronic (14 days) systemic administration would produce similar efficacy against S-IRA. These galanin analogs have a short half-life (1–2 h) (Metcalf et al., 2017a), thereby requiring repeated (multiple times/day) injections, implantable minipumps were used to provide a more consistent compound exposure. Therefore, SC minipumps were prepared to administer daily doses of 4 or 16 mg/kg of 505–5 and 810–2, respectively. Galanin receptors are G-protein coupled transmembrane proteins and develop tolerance to repeated agonist exposure warranted verification of efficacy (Flynn and White, 2015; Lang et al., 2015). Because these analogs are effective in the mouse 6 Hz seizure model (Bulaj et al., 2008; White et al., 2009; Metcalf et al., 2017a), a single stimulus was administered at the end of the study on the day prior to MES testing. As shown in Table 2 for the 6 Hz assay, 505–5 and 810–2 both reduced the number of seizures, with 505–5 producing superior efficacy (21/26 protected, *P* < 0.001 vs. VEH), but 810–2 also proving efficacious (6/11 protected, *P* < 0.05 vs. VEH). On the following day, MES was performed and it was observed that a majority of VEH-treated mice died following TE (75%, 12/16). Also, while 505–5 did not improve mortality from S-IRA (69% mortality, 18/26), 810–2 significantly reduced S-IRA (18%, 2/11, *P* < 0.01 vs. VEH) (Table 2). In the MES assay, neither 810–2 nor 505–5 prevented TE at the doses tested.

**TABLE 2:** Reduced S-IRA following SC administration of galanin analogs in C57Bl/6J mice.

Groups	Dose (mg/kg/day)	6 Hz efficacy (# protected/N)	Mortality (# died/N) (%)
VEH	0	2/16	12/16 (75%)
505–5	4	21/26***	18/26 (69%)
810–2	16	6/11*	2/11** (18%)

*P < 0.05, **P < 0.01, ***P < 0.001 vs. VEH, Fisher’s exact test. A total of 6 Hz testing occurred on the day prior to MES testing and mortality assessment following S-IRA. All mice tested experienced tonic extension.

### Evaluation of post-ictal hypoxia, respiration, and heart rate following maximal electroshock seizure seizures

**FIGURE 1:**
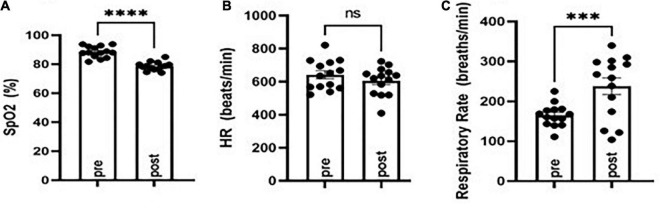
Evaluation of oxygen saturation (SpO_2_), respiratory rate, and heart rate before (pre) and following (post) MES-induced tonic extension in CF-1 mice. SpO_2_ was diminished **(A)** concomitant with an increased respiratory rate **(B)** and no major changes in heart rate **(C)**. ^***^P < 0.001, ^****^P < 0.0001 compared to pre, Student’s *t*-test. *N* = 14.

To study effects on postictal respiration following MES, we evaluated SpO_2_, respiratory rate, and heart rate in CF-1 mice. Notably, this strain of mice survives TE despite having a period of apnea following seizures. VEH-treated CF-1 mice subjected to MES-induced TE experience a brief period (10–20 s) of apnea and decreased SpO_2_, which coincides with tachypnea (see Figure 1). There were no major changes in heart rate observed.

**FIGURE 2:**
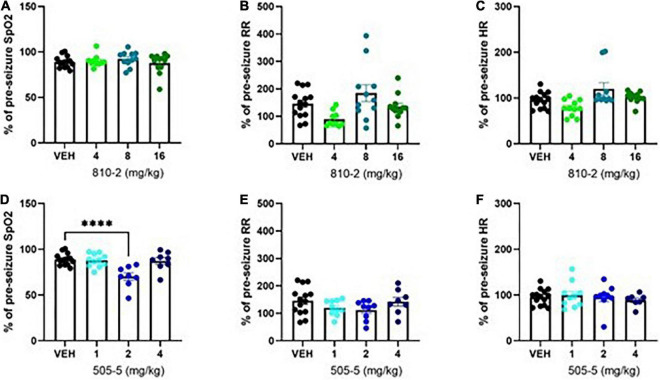
Evaluation of cardiorespiratory parameters following tonic extension in CF-1 mice. Data are presented as a percentage of baseline (pre-seizure) values. VEH-treated values are the same as shown in Figure 1, except expressed as a percent of baseline. A total of 810–2 **(A–C)** and 505–5 **(D–F)** were administered by IP injection 1 h prior to tonic extension and testing (MouseOx). Oxygenation [SpO_2_; **(A,D)**], respiratory rate [RR; **(B,E)**], and heart rate [HR; **(C,F)**] were assessed for all animals. *N* = 8–14. ^****^P < 0.0001 (One-way ANOVA, Sidak’s multiple comparison *post-hoc* test).

Following a baseline MouseOx analysis, galanin analogs were administered by IP injection 1 h prior to testing. VEH, 810–2 (dose range 4–16 mg/kg), and 505–5 (dose range 1–4 mg/kg), were administered prior to a single MES stimulation and 5 min observation using MouseOx. A total of 810–2 was without major effect on post-ictal hypoxia (SpO_2_), tachypnea (respiratory rate), or heart rate (Figures 2A–C). By contrast, 505–5 worsened hypoxia at 2 mg/kg (Figure 2D) but was without major effect on respiratory rate or heart rate (Figures 2E,F).

### Galanin analogs reduce seizure-induced respiratory arrest in fully kindled mice

#### Evaluation of corneal kindling as a potential model of sudden unexpected death in epilepsy

We obtained kindling records from the NIH Epilepsy Therapy Screening Program for several cohorts of CF-1 mice subjected to the corneal kindling paradigm. While this mouse strain is resistant to mortality when experiencing TE under naïve conditions, fully or partially kindled CF-1 mice often die following TE. To determine whether kindling state (fully kindled vs. partially kindled) affected S-IRA, we evaluated multiple cohorts of kindling data. Data presented here are from naïve kindled mice not treated with any investigational compounds. We observed that fully kindled mice have a high mortality rate, particularly when TE occurs after acquiring full kindled status (Supplementary Figure 1).

In separate studies, a cohort of CF-1 mice were kindled and subjected to baseline and post-ictal (MES assay) cardiorespiratory evaluation (MouseOx). We also observed that although SpO_2_ was similarly reduced in age-matched control (CONT) and fully kindled (KIND) mice following TE, respiration did not significantly increase in KIND mice, though this change was present in mice that were partially kindled (PART; i.e., received daily stimulations but failed to acquire full kindled status) (Supplementary Figure 2). Interestingly, heart rate was decreased in KIND mice following TE, whereas it was unchanged in other groups.

#### Evaluation of the effect of galanin analogs on seizure-induced respiratory arrest in fully kindled mice

As a comparator to studies in naïve C57Bl/6J mice, a cohort of mice from this strain was fully kindled and subjected to treatment with galanin analogs using MES-induced TE. Initially, a group of untreated kindled mice was subjected to MES-induced TE and it was observed that a majority (6/9 animals died following TE). Similarly, VEH-treated kindled mice also demonstrate a high mortality after TE (11/16 died) (Table 3). A total of 505–5 improved mortality following TE in kindled mice (4/17 died) (Table 3). While 810–2 had a similar magnitude of effect, the difference was not significant compared to VEH (Table 3).

**TABLE 3:** Reduced S-IRA following a single administration of galanin analogs in fully kindled C57Bl/6J mice.

Groups	Dose (mg/kg)	Mortality (# died/N) (%)
VEH	0	11/16 (69%)
505–5	2	4/17* (24%)
810–2	16	2/8 (25%)

All mice tested experienced tonic extension. *P < 0.05 vs. VEH; Fisher’s exact test.

Table 4 includes a summary of major results observed in these studies. In seizure-naïve mice, systemic (IP, SC mini-pumps) and central (ICV, IA) administration reduced S-IRA, likely due to a Gal_2_ mechanism. Central (ICV) administration, by contrast, appeared to reduce S-IRA predominantly *via* Gal_1_. In seizure experienced animals, the reduction in S-IRA may be due to both Gal_1_ and Gal_2_ (comparable degree of effect for both analogs).

**TABLE 4:** Summary of major findings for the effect of galanin analogs on S-IRA.

Receptor effect	IP	ICV	IA	SC (pump)
	Seizure-naïve			
Gal_1_	±	+	−	−
Gal_2_	+	−	+	+
	Seizure-experienced			
Gal_1_	+	NE	NE	NE
Gal_2_	±	NE	NE	NE

Gal_1_-preferring analog (505–5), Gal_2_-preferring analog (810–2). ND (not evaluated). (+) significant reduction in S-IRA, (–) no differences observed.

## Discussion

SUDEP is a major cause of mortality in epilepsy, and better understanding of mechanisms of death may aid in the development of novel therapies to prevent or reduce the likelihood of death following seizures. Recent studies in human SUDEP have suggested a potential role for the neuropeptide galanin in the amygdala. Therefore, we sought to further evaluate the role of galanin in a mouse model of S-IRA under various conditions. Our principal findings include (1) galanin analogs reduced S-IRA in naïve mice following systemic and central administration, (2) 810–2 prevented mortality following sub-chronic administration, (3) protective effects of systemic galanin analogs were confirmed in fully kindled mice.

Poor adherence to treatment or pharmacoresistance may increase the occurrence of GTCS in patients with various forms of epilepsy. Further, GTCS are a critical risk factor for SUDEP. While genetic and acquired epilepsy models may recapitulate seizures and mortality in epilepsy, drug screening in these models may be challenging due to the need for prolonged administration and monitoring to determine whether treatment reduces seizure burden, TE, and mortality. This kind of study may be critical in demonstrating translational relevance for novel therapies, but throughput may be low. However, acute treatment and testing models may be more amenable to drug screening and help identify lead candidates for more rigorous testing. Therefore, we used our previous knowledge of galanin analogs in S-IRA to better understand potential benefits of galanin-based therapies on reducing the likelihood or onset of SUDEP. Systemic administration of receptor subtype preferring analogs suggested that targeting of Gal_2_ may be beneficial, as 810–2 demonstrated superior reduction in S-IRA over 505–5. Of note, the systemic doses used for each compound were not raised beyond those used in the study, as higher doses are associated with untoward effects (sedation and motor impairment, data not shown). Therefore, future endeavors with galanin analogs may seek to optimize administration through combinatorial pharmacology. For example, recent work has clearly demonstrated a potential benefit for serotonergic therapies in reducing the incidence of SUDEP in animal models (Richerson and Buchanan, 2011; Purnell et al., 2017; Buchanan, 2019; Kruse et al., 2019). While beyond the scope of this study, combined treatment with serotonin- and galanin-based therapies may yield an added benefit.

We sought to extend our systemic administration studies by using central injection to specifically target regions where galanin may exert protective effects against S-IRA. Galanin is expressed throughout the forebrain neurocircuitry, including several sites that affect cardiorespiratory and autonomic function. ICV administration studies suggest an important role for Gal_1_ over Gal_2_ in reducing S-IRA. Conversely, Gal_2_ played a more prominent role when galanin analogs were administered directly into the amygdala. The amygdala has emerged as an important regulatory center for the interaction of seizures with respiratory control. Apnea results when seizures spread to the amygdala and amygdala lesions reduce S-IRA in mice (Marincovich et al., 2019; Nobis et al., 2019; Rhone et al., 2020). Additionally, the amygdala has reciprocal connections with brainstem respiratory centers (Saha et al., 2000; Dong and Swanson, 2006). Importantly, galanin is depleted in the amygdala in human SUDEP (Somani et al., 2020), which suggests that supplemental galanin in this region may reduce the incidence of SUDEP. The reason behind these discrepancies in our ICV and IA data are unclear but may involve multiple neural circuits affecting respiration after seizures. Furthermore, it is important to recognize that IA administration was centered over the basolateral amygdala but may have also spread to the central amygdala. The central amygdala may play an important role in post-ictal breathing by suppressing respiration (Nobis et al., 2018, 2019; Marincovich et al., 2019; Rhone et al., 2020). Future work may include application of galanin analogs in a more subregion-specific and receptor subtype-specific manner.

Tonic extension is lethal in a variety of mouse strains. We evaluated TE following MES stimulation in C57Bl/6J, CD-1, and CF-1 mice. Both C57Bl/6J and CD-1 mice experience similar mortality following MES stimulation. Given this observation, improved mortality following treatment with galanin analogs was the most important outcome observed in these strains. Further, we observed differential responses to galanin compounds: 810–2 (Gal_2_-preferring) was effective in both strains in reducing S-IRA whereas 505–5 (Gal_1_-preferring) was only effective in CD-1 mice. The mechanism for this strain-dependent discrepancy is unknown and may be due to different galanin receptor expression in brainstem respiratory centers between the two species. Future studies may further explore these potential differences. CF-1 mice generally survive TE and therefore this strain was used to study respiration following seizures. Despite the tendency to survive MES stimulation, we observed that this mouse strain demonstrates respiratory distress (diminished SpO_2_, tachypnea) following TE. Thus, TE is a major respiratory stressor, yielding periods of apnea that may be insurmountable for some mouse strains. We hypothesized that galanin compounds may prevent mortality by preventing post-ictal hypoxia and therefore we evaluated oxygenation, heart rate, and respiratory rate in CF-1 mice treated prior to MES stimulation. Neither galanin compound prevented post-ictal hypoxia at any dose tested. Further, 505–5 demonstrated an exacerbation of this response (2 mg/kg). Therefore, the protective effects of galanin compounds in S-IRA may not be due to a direct effect on preventing the cardiorespiratory response to apnea.

G-protein coupled receptors may undergo internalization following stimulation with agonists (Calebiro and Godbole, 2018). To explore the use of galanin compounds as potential therapeutic agents, we performed sub-chronic administration using implanted minipumps pre-filled with either 810–2 or 505–5. To confirm antiseizure efficacy at the doses selected, we first performed 6 Hz seizure testing and observed that both 810–2 and 505–5 retained efficacy in this assay. On the following day, mortality following TE was assessed, and we observed that only 810–2 was effective in reducing mortality. The effect of 810–2 in this assay is consistent with the acute (single administration, IP) effect of this compound and confirms that systemic administration of Gal_2_-preferring compounds exerts a protective effect against S-IRA.

Corneal kindling has long been used as an assay for studying epileptogenesis, epilepsy pathology, and antiseizure drug pharmacology. We observe that in CF-1 mice, a portion of animals die during and following kindling acquisition. We obtained several historical data cohorts from the ETSP (NIH, NINDS) Contract Site (University of Utah) and reviewed kindling histories for the presence of TE after daily kindling stimulation. We reviewed data from naïve kindled mice and we surmised that while naïve CF-1 mice may be resistant to death following TE, this may shift in a kindled state. We identified several animals that died after having experienced one or more bouts of TE. Deaths observed in this cohort did not necessarily occur immediately following TE. Further, these data are consistent with clinical observations that the presence of GTCS are a major risk factor for SUDEP. To better understand the effect of TE on kindled mice, we subjected kindled CF-1 mice to pulse oximetry recordings before and after MES stimulation. We observed that oxygenation drops following TE in kindled mice in a similar manner to age-matched control mice. Interestingly, tachypnea observed in control mice was not observed to the same extent in kindled mice, though heart rate was diminished in these animals. Reduced post-ictal tachypnea among kindled mice may contribute to an increased risk for mortality in some animals.

To extend our studies of galanin analogs as protective of S-IRA in naïve mice, we also evaluated these analogs in fully kindled C57Bl/6J mice. In contrast to observations in naïve mice, where 505–5 was ineffective in reducing S-IRA, 505–5 reduced mortality (2 mg/kg) following TE. These data suggest a potential shift in receptor expression in brain respiratory centers toward Gal_1_ sensitivity (e.g., receptor upregulation or downregulation of Gal_2_), though this was not examined in the present study. Both Gal_1_ and Gal_2_ are expressed in the amygdala (Moller et al., 1999; Lundstrom et al., 2005; Christiansen and Woldbye, 2010; Webling et al., 2012; Li et al., 2017). While galanin receptor changes may occur in epilepsy (e.g., in hippocampus), the relative changes of each of these receptors in the amygdala are unknown. Future studies will examine the expression of each galanin receptor subtype in kindled mice.

Our previous work identified 505–5, 810–2, and other analogs as anticonvulsant neuropeptides in the mouse 6 Hz, Frings audiogenic, and corneal kindling models (Bulaj et al., 2008; White et al., 2009; Bialer et al., 2010, 2013, 2015). It was also observed during initial studies with these analogs that they were ineffective in preventing TE following MES stimulation. These studies, as well as several others examining galanin in seizure models (Mazarati et al., 1992, 1998; Saar et al., 2002; Lundstrom et al., 2005; Mazarati and Lu, 2005; Mitsukawa et al., 2008), provide a strong rationale for the therapeutic potential for galanin analogs as antiseizure medications. Herein we have extended these findings by demonstrating that galanin analogs can prevent S-IRA following TE in naïve and seizure experienced animals.

### Limitations of the study

It is noteworthy that only males were used in screening for the reduction of S-IRA in these studies. Although seizure thresholds for TE are similar between males and females (data not shown), it is possible that the pharmacological response and/or prevention of mortality may be different in female animals. Future studies may extend these observations to more etiologically relevant animals models consisting of spontaneous seizures, wherein both male and female animals can be studied. Central injections were used for galanin analogs based on previously established protocols for ICV (Lee et al., 2009; Green et al., 2011; Platt et al., 2012) and IA (West et al., 2022) injections. However, verification of injection location was not performed in the current studies. It is possible that improper ICV injection and local diffusion rather than ventricular distribution may account for the effects observed. Further, IA injection not properly administered may have affected surrounding regions that also control respiration (Homma and Masaoka, 2008; Evans, 2010; Trevizan-Bau et al., 2021). Because central injection parameters were not verified explicitly, it is possible that off-target effects arising from galanin analog administration may have contributed to the outcomes observed. The precise mechanism whereby galanin agonism may prevent S-IRA has yet to be determined. While galanin may play an important modulatory role on synaptic transmission in key respiratory control centers such as the amygdala, the means whereby galanin may protect against S-IRA is unclear. Future studies may include the use of a neuronal activation marker (e.g., Fos) following treatment and stimulation to determine the effect of galanin analogs on post-ictal activation of respiratory cell populations.

In summary, we have observed that galanin compounds protect against S-IRA following TE in naïve and kindled mice. This was observed after systemic and central administration, and responses varied depending on route, location, strain, seizure history, and receptor subtype preference. While these studies suggest the potential translational benefit for galanin in SUDEP prevention, additional work is needed to differentiate the utility of these analogs in SUDEP-susceptible individuals.

## Data availability statement

The datasets presented in this article are readily available and can be accessed by contacting CM (cameron.s.metcalf@utah.edu).

## Ethics statement

This animal study was reviewed and approved by the Institutional Animal Care and Use Committee, University of Utah.

## Author contributions

RC designed and conducted the experiments, analyzed the data, and prepared the manuscript. MA and ED designed and conducted the experiments and analyzed the data. KR and CC conducted the experiments. CM designed and conducted the experiments, analyzed the data, and prepared the manuscript, oversaw all work. All authors contributed to the article and approved the submitted version.
